# Diagnosis and Management of a Prolapsing Intravesical Ureterocele in a Man

**DOI:** 10.1089/cren.2015.29001.der

**Published:** 2015-10-01

**Authors:** Samir Derisavifard, Piruz Motamedinia, Zeph Okeke, Arthur Smith

**Affiliations:** The Smith Institute for Urology, North Shore-Long Island Jewish Health System, New Hyde Park, New York.

## Abstract

Given the low incidence and delayed diagnosis, ureterocele management in the adult population is poorly described in the literature. Moreover, there is only one case report characterizing the condition with prolapse in an adult male. Approaches to therapy include transurethral incision or puncture with or without a combined percutaneous approach, and excision with or without partial nephrectomy for a duplicated system with a nonfunctioning upper pole moiety. We present a case of prolapsed, single intravesical ureterocele in a man presenting with hematuria and lower urinary-tract symptoms. A 54-year-old man with no significant medical history presents with increasing nocturia and urinary hesitancy. The development of gross hematuria prompted urologic evaluation. On imaging, the patient was found to have 4.3 × 3.3 cm bladder mass consistent with a prolapsed ureterocele that was managed by transurethral excision with a cutting loop. Postoperatively, the patient's symptoms resolved completely without complication. We suggest that complete transurethral ureterocele excision is an effective, definitive treatment option.

## Clinical History

A 54-year-old man with a history of only hyperlipidemia presented with urinary hesitancy and nocturia four to five times for ∼3 years. More recently, his symptoms were associated with hematuria, which prompted urologic consultation. He denied hematuria on the day he presented for evaluation. He denied any history of stranguria, dysuria, or incomplete emptying. In addition, the patient is a nonsmoker and denied a history of environmental exposures. He had no family or personal history of genitourinary cancer or nephrolithiasis.

## Physical Examination and Diagnosis

**FIG. 1.**
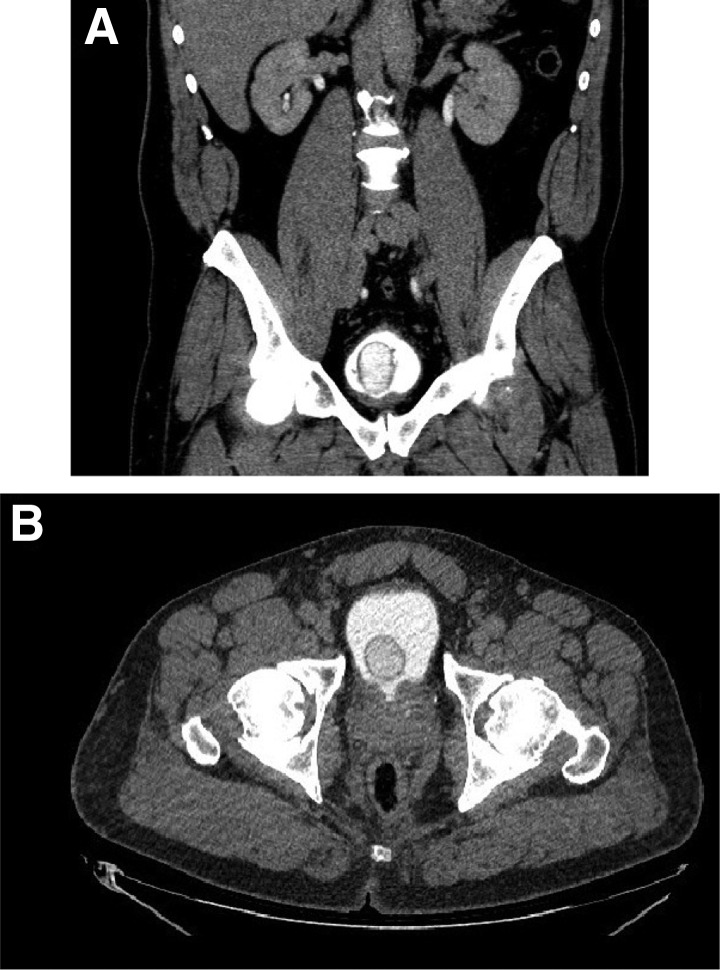
Computed tomography scan with intravenous contrast of the abdomen and pelvis; delayed imaging. Coronal cross-sectional imaging shows the right ureterocele within the bladder **(A)**. Transverse imaging demonstrates the proximity of the ureterocele to the bladder neck and outlet **(B)**.

On examination, the patient had a nontender nonnodular prostate that was not enlarged, and was without abdominal or costo-vertebral angle tenderness. Urinalysis at the time of evaluation revealed no red blood cells and was negative for nitrites and leukocyte esterase. Urine culture was additionally negative. Computed tomography with intravenous contrast and delayed-phase images indicated a large 4.3 × 3.3 cm round filling defect within the bladder (Fig. 1). The mass filled with contrast, suggesting a ureterocele. Interestingly, there was no hydronephrosis or delay in the ipsilateral nephrogram that would have otherwise suggested upper tract obstruction.

## Intervention

Given the patient's complaints of hematuria and voiding symptoms, endoscopic incision of the ureterocele was suggested for definitive management. Cystourethroscopic visualization of the ureterocele under anesthesia revealed a normal urethra, mild prostate enlargement with erythematous prostatic lobes, and a bladder neck indicative of chronic inflammation. A large edematous mass at the bladder neck consistent with a ureterocele was easily identified. However, the contralateral ureteral orifice was only identifiable with retroflexion of the flexible cystoscope and lateral displacement of the ureterocele. After filling the bladder and retraction of the cystoscope into the urethra, ureterocele prolapsed into and partial obstruction of the prostatic urethra with manual crede were noted.

Our decision to excise rather than merely puncture was based on the bladder neck obstruction and irritation by the mass rather than any signs of ureteral obstruction. This differs from the documented ureterocele incision with a transcutaneous approach in a male of similar age.^1^ We believed that given the moderate ureterocele wall thickness, a simple puncture would likely not allow for adequate decompression and involution of the ureterocele and our objectives would not be met; and so, monopolar cutting loop was used to make a circumferential incision around the base of the ureterocele, which allowed for complete excision. The residual ipsilateral ureteral orifice was noted to be orthotopic, of normal caliber, and surrounded by a normal urothelium. Resultantly, a ureteral stent was deemed unnecessary. This technique thus offers an additional approach to ureterocele treatment that augments the armamentum of the surgeon in adding to other incisional and percutaneous approaches characterized in the literature.^2,3^

## Follow-Up

**FIG. 2.**
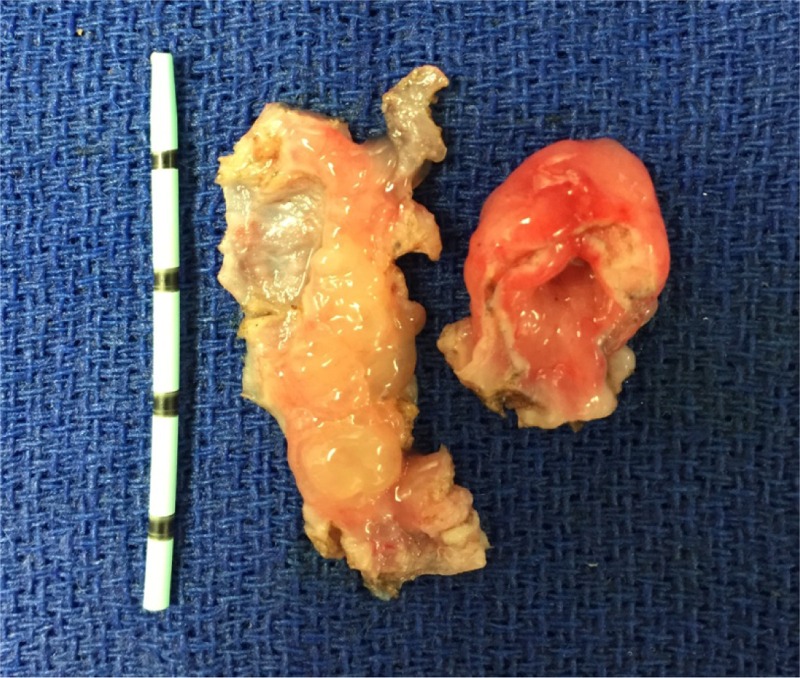
Ureterocele, gross specimen. The specimen on the *left* is the circumferential base of the ureterocele spatulated open to demonstrate the inflamed mucosal inner lining. The specimen on the *right* illustrates the relatively thick wall of the ureterocele.

**FIG. 3.**
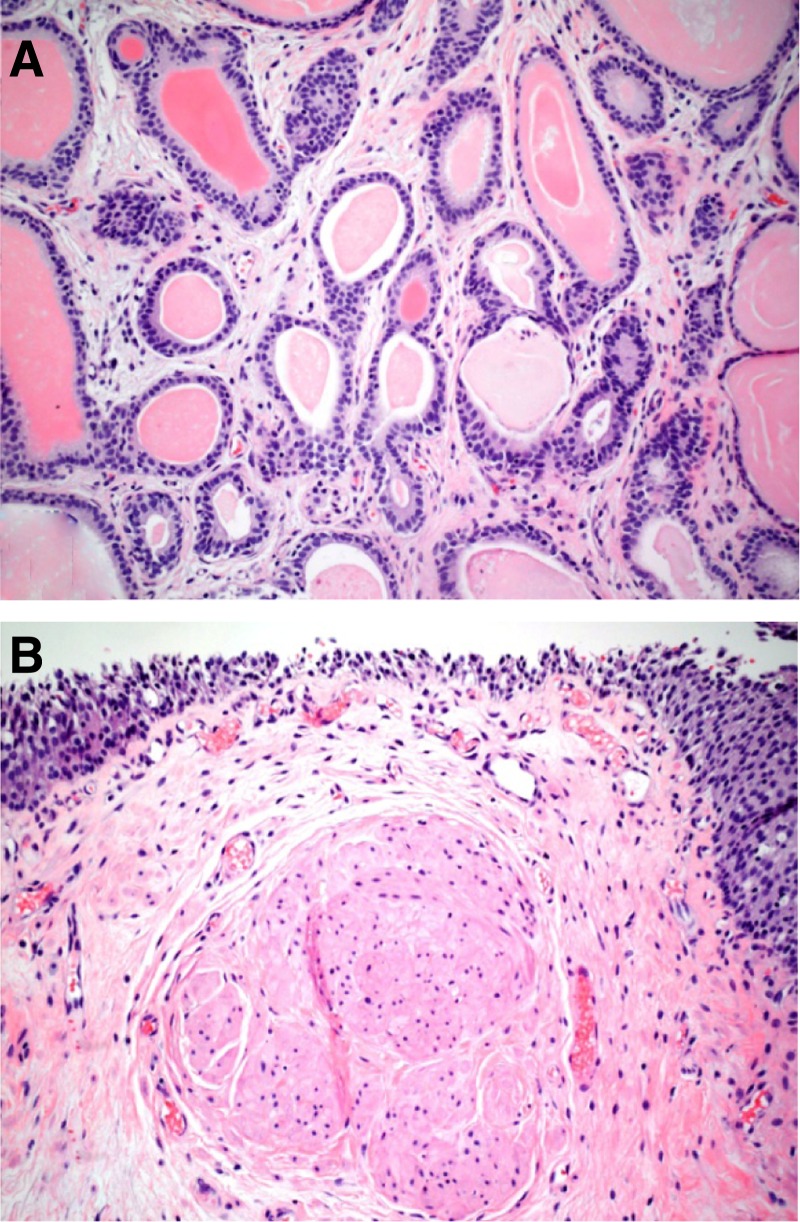
Ureterocele, microscopic specimen. Florid ureteritis cystic **(A)**. Benign urothelial cells overlying lamina propria and the distinct muscle fibers of muscularis propria **(B)**.

The patient was discharged home the same day. On postoperative follow-up, the patient stated that he had immediate improvement of urinary symptoms and a strong stream. Pathologic assessment of the ureterocele was relevant for a hollow mass, 3 cm in its largest diameter with a thick wall (Fig. 2). Microscopic analysis demonstrated a benign urothelium with ureteritis cystica (Fig. 3A) and definite muscularis propria (Fig. 3B). There was no evidence of malignancy.
